# C-terminal interactions mediate the quaternary dynamics of αB-crystallin

**DOI:** 10.1098/rstb.2011.0405

**Published:** 2013-05-05

**Authors:** Gillian R. Hilton, Georg K. A. Hochberg, Arthur Laganowsky, Scott I. McGinnigle, Andrew J. Baldwin, Justin L. P. Benesch

**Affiliations:** Department of Chemistry, Physical and Theoretical Chemistry Laboratory, University of Oxford, South Parks Road, Oxford OX1 3QZ, UK

**Keywords:** mass spectrometry, small heat-shock protein, molecular chaperone, subunit exchange, allostery

## Abstract

αB-crystallin is a highly dynamic, polydisperse small heat-shock protein that can form oligomers ranging in mass from 200 to 800 kDa. Here we use a multifaceted mass spectrometry approach to assess the role of the C-terminal tail in the self-assembly of αB-crystallin. Titration experiments allow us to monitor the binding of peptides representing the C-terminus to the αB-crystallin core domain, and observe individual affinities to both monomeric and dimeric forms. Notably, we find that binding the second peptide equivalent to the core domain dimer is considerably more difficult than the first, suggesting a role of the C-terminus in regulating assembly. This finding motivates us to examine the effect of point mutations in the C-terminus in the full-length protein, by quantifying the changes in oligomeric distribution and corresponding subunit exchange rates. Our results combine to demonstrate that alterations in the C-terminal tail have a significant impact on the thermodynamics and kinetics of αB-crystallin. Remarkably, we find that there is energy compensation between the inter- and intra-dimer interfaces: when one interaction is weakened, the other is strengthened. This allosteric communication between binding sites on αB-crystallin is likely important for its role in binding target proteins.

## 1. Introduction

αB-crystallin is an oligomeric vertebrate small heat-shock protein (sHSP) with molecular chaperone activity [1]. In line with the general mechanism of action suggested for the sHSPs [2–5], αB-crystallin can trap non-native proteins in such a manner that prevents their aggregation [6] and facilitates their recovery by the downstream ATP-dependent molecular chaperone machinery [7]. Furthermore, αB-crystallin has been shown to bind to mature amyloid fibrils, inhibiting their elongation [8–11]. These functions represent an important mechanism that enables the cell to cope with the burden of unfolded proteins and maintain ‘proteostasis’ [12–14]. It is therefore no surprise that malfunction of αB-crystallin has been linked to numerous protein deposition diseases ranging from cataract formation [15] to motor neuropathies [16] and neurodegenerative disease [17].

**Figure 1.**
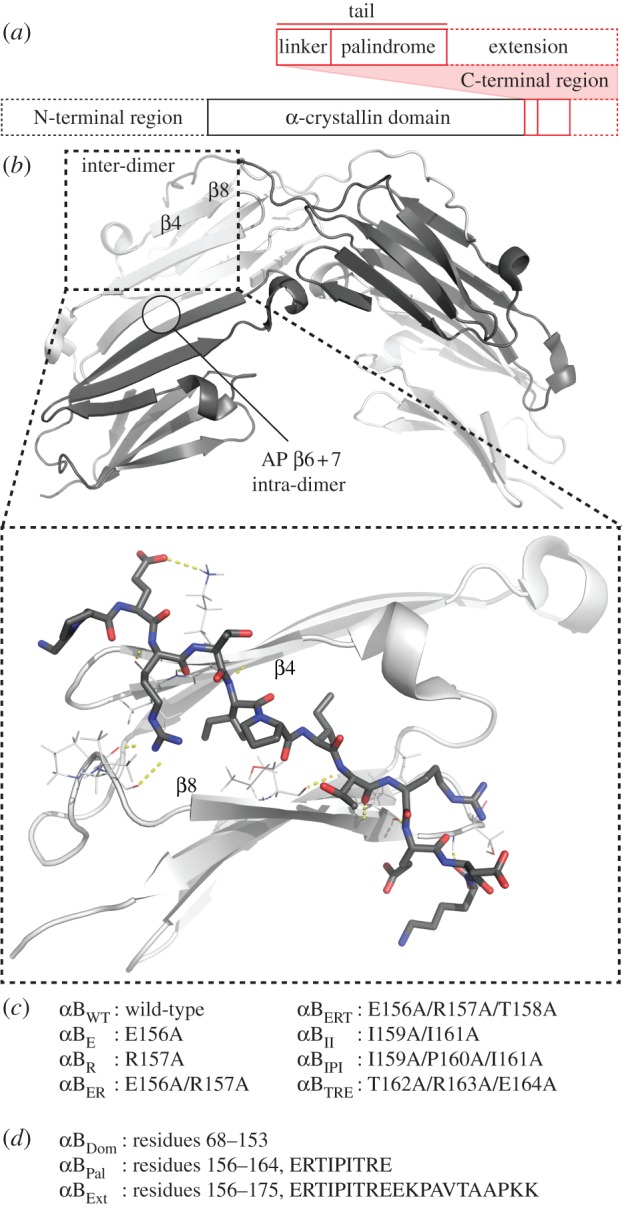
(*a*) The primary sequence of αB-crystallin consists of N- and C-terminal regions flanking a core ‘α-crystallin’ domain. The C-terminal region is itself split into three sections. A palindromic region of sequence, centred on an IXI motif that is highly conserved amongst sHSPs, is preceded by a linker to the α-crystallin domain. Combined, these two sections are often termed the C-terminal ‘tail’, and are followed by the ‘extension’, a region of sequence that is highly flexible. Atomic resolution information is lacking for the extension, as well as the N-terminal region (dashed lines). (*b*) Crystal structure of a truncated construct of αB-crystallin (PDB: 3L1G) illustrating two types of inter-molecular contacts: an intra-dimer interface between two β6+7 strands, and an inter-dimer interface between the C-terminal tail and a hydrophobic groove separating the β4 and β8 strands on an adjacent monomer. The expansion highlights this latter interaction (for a modelled peptide, see §2*e*), showing the palindromic residues 156–164 and the hydrogen bonds (yellow dashes) made with the groove. (*c*) The nomenclature used for all the point mutants of full-length αB-crystallin, and (*d*) truncated domain and C-terminal peptides under investigation here is tabulated. (Online version in colour.)

Despite several thousand sHSP genes having been deposited in the UniProt database, there is relatively scarce detailed information about their assembled structures [2–5]. This is primarily due to their dynamic and frequently polydisperse nature, properties that make high-resolution studies particularly challenging [18]. αB-crystallin assembles into an ensemble of interconverting oligomeric states spanning approximately 10–40 subunits [19,20]. In recent years, considerable strides have been made in overcoming this heterogeneity and interrogating the structure of αB-crystallin. X-ray crystallography studies of N- and C-terminally truncated constructs of both αB-crystallin and its eye-lens-specific isoform αA [21–24] as well as solid-state nuclear magnetic resonance spectroscopy (ssNMR) of the full-length protein [25] have revealed the structure of a dimeric ‘building block’. This protomer is composed of a β-sandwich ‘α-crystallin’ core that assembles through anti-parallel (AP) pairwise interactions between extended β6+7 strands (figure 1). This interface appears common to metazoan sHSPs, and has been observed in different registration states [21–24], termed AP_I_, AP_II_ and AP_III_ [23].

αB-crystallin dimers assemble into oligomers via interactions mediated, at least in part, by the terminal regions [4,15]. Although few spatial restraints have been obtained to define full structural details of their contribution, the N-termini clearly provide some stabilization of the oligomers [26,27]. Removal of this region shifts the equilibrium towards sub-oligomeric proteins, but nevertheless, the oligomerization competency of the protein is retained [23]. Crystal structures of the related HSP16.5 [28], HSP16.9 [29] and HSP14.0 [30] oligomers reveal their C-termini to span between dimers, such that a highly conserved IXI motif can bind to a hydrophobic groove between β4 and β8 strands. A similar inter-dimer interaction mediated by the C-termini has also been observed experimentally in αB-crystallin [23,25,31,32], and has been explicitly used to guide the modelling of putative oligomer structures [26,27,33].

Precise molecular details of the C-terminal residues of αB-crystallin, however, remain the subject of some contention [4,15]. The sequence of this region can be considered as separate segments, the ‘tail’ comprising residues up to and including a palindromic motif, and the ‘extension’ being all residues downstream (figure 1*a*) [4]. The C-terminal extension is typically disordered and tumbles freely in solution [34]. The crystal structure of a construct of αB-crystallin, truncated of the extension in addition to the N-terminal region, formed a runaway domain-swapped polymer, with the IXI lodged in the β4–β8 groove (figure 1*b*) [23]. This binding of the IXI was also demonstrated to pertain to the full-length protein in ssNMR performed at low temperature [25]. By contrast, NMR experiments performed in solution at physiological temperatures and pH revealed the IXI in full-length αB-crystallin to primarily populate an intrinsically disordered conformation [31,35–37]. The apparent contradiction between these studies has recently been rationalized by a temperature-dependent transition between conformations: a mixture of both bound and unbound states were observed below 0°C (in both solution and ssNMR experiments), whereas at temperatures above this only the unbound state was readily observed [36].

Fluctuations in the C-terminus on the millisecond time-scale have been shown to be rate-limiting in the movement of subunits between αB-crystallin oligomers [31]. Moreover, removal of residues from the flexible C-terminal extension has been shown to reduce the rate of this subunit exchange [38,39]. Considering the tight regulation of αB-crystallin dynamics by residues in this region of the protein, and the seemingly crucial role of plasticity in sHSP function [2–5], it is perhaps not surprising that mutations near the C-terminus display aberrant molecular chaperone function *in vitro* [32,40], in cells [41,42] and are associated with disease [43–46].

In this study, we investigate the molecular details of the interaction between the α-crystallin C-terminal region and the β4–β8 groove in the core domain. We perform our investigations using nanoelectrospray mass spectrometry (MS) and ion-mobility spectrometry (IM). Since its initial applications to the study of protein assemblies in the early 1990s [47], MS has matured as a structural biology approach to allow the accurate determination of oligomeric stoichiometry and dynamics [48–50]. We exploit these benefits here to characterize in detail the influence of residues in the C-terminal tail of αB-crystallin, ^156^ERTIPITRE^164^, which is highly conserved as a palindrome throughout the α-crystallin family [23]. We first investigate the binding of these amino acids, in the form of a peptide, to the isolated αB-crystallin core domain and extract the associated association constants. Using this approach, we find that the C-terminal extension destabilizes binding of the IXI motif, preventing it from forming the more tightly bound conformations observed in other sHSPs. To extend this work to the context of the full-length protein, we describe a series of point mutations to alanine (figure 1*c*), and characterize their effect on the oligomeric distribution and subunit exchange dynamics of the protein. We find that mutations in the C-terminal tail significantly affect the stability and dynamics of the oligomers, indicating that, while predominantly disordered, they are nonetheless able to exert significant influence on determining which oligomers are populated. We note that mutations of pseudo-equivalent palindrome residues do not display equivalent changes in thermodynamics and kinetics, suggesting a favoured orientation within the oligomers. Moreover, we observe a strong negative correlation between the free energy of the inter- and intra-dimer interface, revealing an allosteric coupling between them.

## 2. Materials and methods

### (a) Protein expression and purification

Full-length αB-crystallin, the C-terminal mutants and a truncated form (residues 68–153, αB_Dom_) were expressed in *Escherichia coli* and purified as previously described [23,51]. The specific mutations in the full-length protein are shown in figure 1 and in the electronic supplementary material, table S1. The peptide, ERTIPITRE, was expressed and purified from *E. coli* as described previously [52], whereas peptides ERTIPITREEKPAVTAAPKK and TIERPREIT were purchased from BioMatik (Canada). Unless otherwise stated, solutions of full-length αB-crystallin were prepared at a monomeric concentration of 20 µM in 200 mM ammonium acetate pH 6.9 prior to MS analysis.

### (b) Measuring peptide binding to the αB-crystallin core domain

All titration experiments were carried out at room temperature and allowed to equilibrate for 30 min prior to measurement. Solutions of αB_Dom_ were prepared at a final monomeric concentration of 4 µM (as determined using a bicinchoninic acid assay on a stock solution) in 200 mM ammonium acetate pH 6.9, whereas the final concentrations of the C-terminal peptides were varied. All C-terminal peptides were dissolved in water, with final concentrations of 4, 8, 16, 32, 64, 128 μM.

Titration data were collected on a Synapt G1 HDMS instrument (Waters Ltd., UK), modified to incorporate a linear drift tube [53]. Parameters were set to minimize activation of gas-phase ions and maximize separation between monomeric and dimeric species in the arrival-time dimension. To ensure the preservation of the labile interaction between peptide and αB_Dom_ in the mass spectrometer, and reduce the number of alkali metal adducts, a small reservoir of acetonitrile was maintained in the source region of the mass spectrometer [54]. The instrumental voltages used were capillary (1400), sampling cone (10), extraction cone (1), trap (5), bias (20) and drift tube (50). The gas flow rates were trap (argon, 2.6 ml min^−1^), drift tube (nitrogen, 50 or 20 ml min^−1^), and the ‘backing’ pressure was 3.8 mbar. Three spectra were recorded for each titration point, using a new needle each time to account for needle-to-needle variation.

### (c) Ion-mobility mass spectrometry titration data analysis

External calibration of the spectra was performed using MassLynx software (Waters Ltd.), and the data were exported for analysis using our home-built algorithm CHAMP [55]. CHAMP was adjusted to incorporate the arrival-time dimension, along the lines we have described previously [33]. The collisional cross-section (CCS) of each molecular species was considered to be invariant with charge state, an assumption that appears valid for protein assemblies in the absence of peturbants [56]. To ensure the stability of the protein assemblies in the gas phase, and CCSs close to those expected from their solution structure, we examined them in a charge-reduced form [57], achieved here by a partial pressure of acetonitrile in the source region [54]. Individual IM–MS peaks were considered to have Gaussian profiles in the arrival-time dimension [33], and asymmetric Lorentzian profiles in the mass-to-charge (*m*/*z*) dimension [58]. The width of the IM peaks was fitted by assuming the full-width-at-half-maximum scales linearly with drift time for ions of similar mobility [59]. Minimization was performed using a combination of stimulated annealing and Levenberg–Marquardt methods [55].

### (d) Determination of peptide binding affinity

To determine the binding affinities of the C-terminal peptides to αB_Dom_, we used the abundances extracted by CHAMP as described earlier. In order to obtain reliable binding affinities, it is important to account for ‘false-positive’ complexes [60]. These arise from non-specific association during the electrospray process, and can become significant at high protein or ligand concentrations [61]. To address this, we performed two sets of control experiments to assess the extent of non-specific binding. In the first, we obtained spectra of the ‘scrambled’ C-terminal peptide TIERPREIT incubated at a range of concentrations with αB_Dom_, and in the second, the specific peptide ERTIPITRE with the unrelated protein cytochrome c. In both cases, the abundance of protein–ligand complexes can be entirely ascribed to non-specific association. In this way, the abundances of detected protein–ligand complexes can be corrected to reflect solely the specific associations [62]. Both these control experiments were found to correspond to an apparent *K*_D_ on the order of millimolar, see the electronic supplementary material. Thus adjusted, the experimental distribution of apo- and holo-αB_Dom_ was examined in terms of a protein–ligand binding model, described in detail in the electronic supplementary material, in order to obtain the binding affinities.

### (e) Assessment of residue-specific C-terminal binding *in silico*

A model structure for *ΔΔG* calculations was constructed from the truncated human αB-crystallin crystal structure (PDB: 3L1G) with the C-terminal tail bound [23] and further modified using atomic coordinates from the bound tail in the structure of truncated zebrafish αA-crystallin (PDB: 3N3E) [24]. Atomic coordinates from 3L1G for residues 68–153 and residues 153–162 of the bound C-terminal tail from a symmetry-derived molecule were merged into one chain. The C-terminal tail was modified by grafting residues 163–166 of 3N3E after alignment of the tail. This model was relaxed using Rosetta v. 3.3, prior to estimating *ΔΔG*s upon mutation *in silico* to alanine [63,64].

### (f) Determination of the oligomeric distributions and interface free energies

All samples were pre-incubated at 37°C for 30 min prior to mixing to ensure they had reached equilibrium. MS was performed under activating conditions as described previously [65]. The resulting spectra can be unambiguously ascribed to the oligomeric distribution in solution owing to the predictable dissociation pathway of protein assemblies in the gas phase [66]. The relative abundances of all the doubly stripped oligomers were quantified from the intensity of the peaks [19], and fitted to an oligomerization model we have described in detail elsewhere [65] to obtain accurate measurements of the free energy of the inter- and intra-dimer interfaces, *ΔG*_e_ and *ΔG*_d_, respectively (*ΔG*_e+d_ = *ΔG*_e_+*ΔG*_d_) (see the electronic supplementary material, table S1). In order to compare the relative effects of the mutations in their various positions, the free energies were considered as *ΔΔG*s according to *ΔΔG* = *ΔG*_mutant_−*ΔG*_WT_.

### (g) Monitoring quaternary dynamics of αB-crystallin

**Figure 2.**
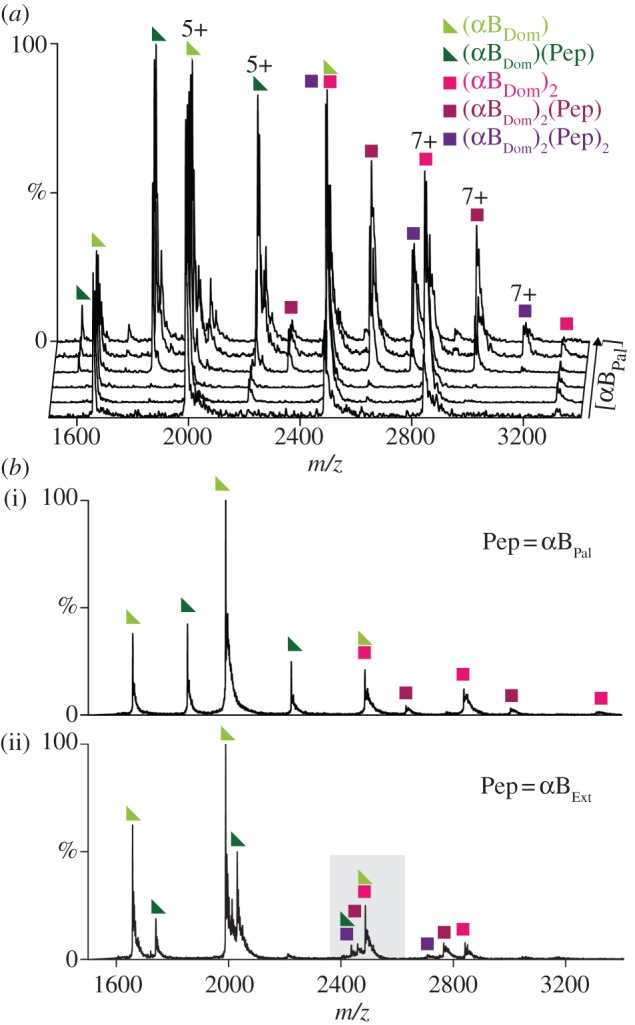
(*a*) Nanoelectrospray mass spectra of 5 μM αB-crystallin ‘core-domain’, αB_Dom_ (comprising residues 68–153), incubated with a peptide αB_Pal_, representing the palindromic C-terminal tail (residues 156–164). The concentrations of αB_Pal_ are 4, 8, 16, 32, 64, 128 μM from front to back. Both monomeric (triangles) and dimeric (squares) αB_Dom_ are visible and, as the concentration of αB_Pal_ is increased, multiple complexes with ligand appear. Up to one peptide is observed to bind the αB_Dom_ monomer, whereas up to two are seen to bind the αB_Dom_ dimer. (*b*) Comparison of spectra obtained at a peptide concentration of 16 μM for αB_Pal_ (i) and αB_Ext_ (ii), with the latter, longer peptide representing the combined tail and extension of αB-crystallin (residues 156–175). Binding is observed in both cases, but quantification of the relative abundances is hampered by the overlap of charge states from the different species (e.g. grey shaded box).

Subunit exchange experiments were performed by incubating wild-type and mutant forms of αB-crystallin with the ^13^C isotopically labelled αB_ERT_, with the exception of WT protein that was mixed with its labelled equivalent. All samples were pre-incubated individually at 37°C for 30 min to ensure they had reached an equilibrium oligomeric distribution prior to mixing at a 1 : 1 ratio. Aliquots of the mixture were taken at various time points, with the reaction quenched on ice, and analysed off-line as described previously [65]. The peak corresponding to all oligomers, each carrying the same number of charges as subunits (i.e. [αB*_x_*]*^x^*^+^; figure 2), enable the time-dependent disappearance of homo-oligomers, and concomitant emergence of hetero-oligomers, to be readily monitored [38,67]. The data were then fitted to our oligomerization model [65], allowing the rate constants of dissociation *k^−^*_e_ and *k*^−^_e + d_, and rate of association *k*^+^[αB_1_] to be extracted (see the electronic supplementary material, table S1).

## 3. Results

### (a) The C-terminal tail of αB-crystallin bind the core domain only weakly

To examine the binding of the tail residues of αB-crystallin, we investigated the binding of peptides to a truncated construct αB_Dom_, residues 68–153, in which the β4–β8 groove is necessarily unoccupied. We performed titration experiments in which we incubated αB_Dom_ with either the palindromic peptide ERTIPITRE (αB_Pal_), representing residues 156–164 of αB-crystallin, or the peptide ERTIPITREEKPAVTAAPKK (αB_Ext_), residues 156–175, the combined C-terminal tail and extension (figure 1*d*).

At the lowest concentration of peptide (4 μM), peaks are observed corresponding to αB_Dom_ monomer and dimer (figure 2*a*). The preponderance of monomer is in line with a weak intra-dimer interface [68] and *K*_D_ in the low micromolar range [23]. Upon increasing the concentration of peptide, peaks appear that correspond to the complexes formed between the protein and peptide at stoichiometries of (αB_Dom_)_1_(αB_Pal_)_1_, (αB_Dom_)_2_(αB_Pal_)_1_ and (αB_Dom_)_2_(αB_Pal_)_2_ (figure 2*a*). Notably, even at the highest peptide concentrations for which we were able to obtain good mass spectra (128 μM peptide, more than a 10-fold excess), we never observed more than about half of the available sites to be occupied. Analogous experiments with αB_Ext_ showed apparently similar levels of bound forms (figure 2*b*). Binding of the C-terminal peptide therefore appears to be relatively weak, in agreement with previous data [31,36,37,69].

### (b) Ion-mobility spectrometry—mass spectrometry allows quantitative extraction of peptide binding affinities to the αB-crystallin core domain

While these MS data show qualitatively that the peptides bind to αB_Dom_, they are challenging to interpret quantitatively owing to the overlap of charge states from the different species, for example (αB_Dom_)^4+^ and (αB_Dom_)_2_ ^8+^. This is particularly noticeable in the case of the cluster of peaks around 2470 *m*/*z* for αB_Dom_ incubated with αB_Ext_ (shaded, figure 2*b*). In order to facilitate the deconvolution of these peaks, we used a hybrid IM–MS strategy. These experiments provide an orthogonal dimension of separation to *m*/*z* according to the ions' ability to traverse a tube of neutral gas at low pressure under the influence of a weak electric field. The transit time through the drift cell of the ions is proportional to their CCS and inversely proportional to charge [70].

**Figure 3.**
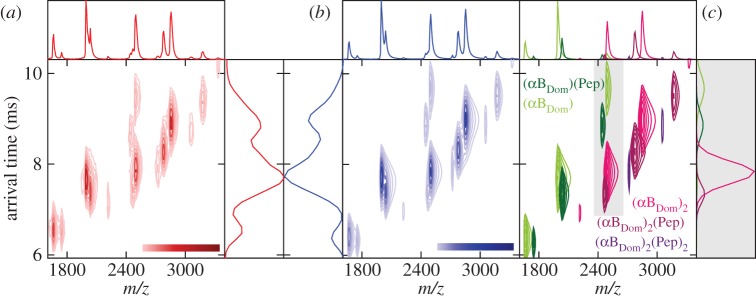
(*a*) Two-dimensional IM–MS spectrum of αB_Dom_ incubated with αB_Ext_ at a concentration of 32 μM, with the corresponding line-projections in the *m*/*z* and arrival-time dimensions. The one-dimensional data are complex, and charge states can be seen to overlap in either dimension. They are however well separated in the two-dimensional IM–MS spectra. (*b*) The data are analysed by determining the best-fitting calculated spectrum using the CHAMP algorithm. The fit (blue) matches the experimental data (red) extremely well, and can therefore be used to obtain the relative contribution of the different species to the overall spectrum. (*c*) A breakdown of the fitted spectrum showing the specific contributions of the monomeric (greens) and dimeric (magentas) species. The arrival-time projection (right panel) shows the projected intensity of the peaks in the range 2350–2650 *m*/*z* (grey shaded box), as in figure 2*b*. The complexity of this region emphasizes the utility of IM for obtaining robust abundances of the various species observed over the course of the titration.

An IM–MS spectrum of αB_Dom_ incubated with αB_Ext_ shows a series of charge states separated in both *m*/*z* and arrival time (figure 3*a*). The separation between monomer and dimer is dramatically improved relative to the one-dimensional MS experiment (figure 2). In order to analyse this spectrum, we used an extension of our spectrum calculation algorithm CHAMP [55], modified to accommodate the arrival-time dimension [33]. By calculating different IM–MS spectra from candidate distributions of the molecular components in the spectrum, and comparing them to the data, a best-fit spectrum is obtained (see figure 3*b* and electronic supplementary material, figure S1). The correspondence between data and fit is excellent, allowing us to deconvolve the relative contributions of the different stoichiometries to the overall spectrum (figure 3*c*).

**Figure 4.**
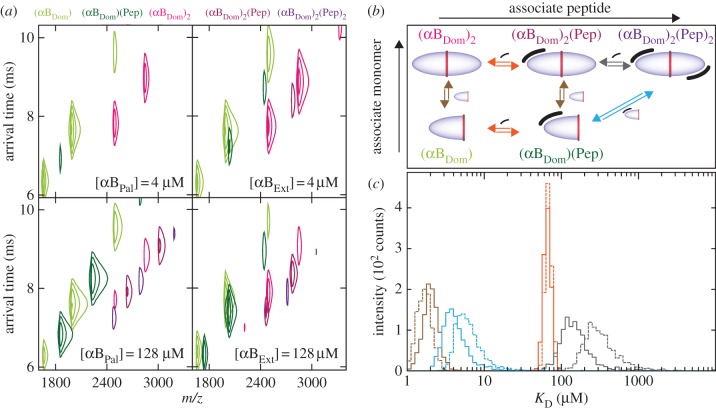
(*a*) Representative IM–MS spectra of αB_Dom_ incubated with either αB_Pal_ (left panels) or αB_Ext_ (right panels). At 4 μM peptide (upper panels), both αB_Dom_ monomers and dimers are observed, with very little peptide binding, revealing that the *K*_D_ must be significantly larger than 1 μM. At 128 μM peptide (lower panels), both apo and holo forms are observed. Notably, the abundance of (αB_Dom_)_2_(Pep)_2_, the domain dimer with two peptides, is less abundant in the case of αB_Ext_ than αB_Pal_, whereas the quantities of (αB_Dom_)_1_(Pep)_1_ are similar in both. (*b*) Quantitative analysis of peptide binding. We analyse the MS titration data in terms of a ligand binding model invoking a total of six coupled equilibria (see the electronic supplementary material for further details) with an increasing number of free parameters. In the simplest model all sets of monomer/dimer equilibria (brown, grey) and peptide binding equilibria (orange, blue) are identical so that the system is controlled by only two independent parameters. Allowing the second peptide binding to have different binding affinity to the first binding event leads to a model specified by four affinities (orange, blue, brown and grey), determined from three independent fitting parameters. The inclusion of this additional parameter is statistically justified for the titrations with αB_Pal_ and αB_Ext_, whereas models of increased complexity were found not to be. As described in the text, these data enable us to conclude that the second peptide binds with lower affinity than the first. The raw data and fits are shown in the electronic supplementary material, figure S1. (*c*) A bootstrap analysis was performed to assess the uncertainty in our estimates of dissociation constants as described in detail in the electronic supplementary material. The resulting histograms are shown for fitting the statistically justified four *K*_D_ model to data from αB_Pal_ (solid lines, colours as in *b*) and αB_Ext_ (dashed lines, colours as in *b*). The histograms reveal that a single peptide has a similar affinity to αB_Dom_ in both cases (orange). The affinity of binding the second peptide to αB_Dom_ is lower for αB_Ext_ than αB_Pal_ (grey). Moreover, the *K*_D_ of (αB_Dom_)_2_(Pep)_2_ is notably higher in the case of αB_Ext_ than αB_Pal_, indicating weaker binding.

Using this approach, we analysed our titration series in order to interrogate the concentration dependence of peptide binding (see the electronic supplementary material, figure S1). At the lowest concentration of peptide (4 μM), peaks are visible corresponding to αB_Dom_ monomers and dimers, and negligible binding of peptide is observed for either αB_Pal_ or αB_Ext_ (figure 4*a*, upper panels). At the highest concentration (128 μM) however, significant abundances of the bound forms are present (figure 4*a*, lower panels). It is notable how in the case of αB_Ext_ significantly less (αB_Dom_)_2_(Pep)_2_ is observed than for αB_Pal_.

To interpret these differences, we examined the abundances of the different molecular species in the context of a protein–ligand binding model (figure 4*b*). In order to adequately reproduce the data, we had to employ a scheme that allowed the second peptide to bind a dimer with an equilibrium constant that differed to that of binding the first (see the electronic supplementary material). For both αB_Pal_ and αB_Ext_, we find that the *K*_D_ of binding the first peptide equivalent was found to be approximately 70 μM, with a dimer interface *K*_D_ of approximately 2 μM (orange and brown, respectively, figure 4*b*,*c*). Remarkably, the affinity for the second peptide equivalent we find to be substantially lower: 150 μM for αB_Pal_ and 300 μM for αB_Ext_ (grey, figure 4*b*,*c*) Furthermore, the dimer interface for (αB_Dom_)_2_(Pep)_2_ is slightly weaker (4 μM for αB_Pal_ and 6 μM for αB_Ext_) than in the other dimeric species. Taken together, our data demonstrate that binding two peptides to a dimer is substantially more difficult than binding just one, and has the concomitant effect of actively weakening the intra-dimer interface. This destabilization effect of the second peptide is substantially greater for αB_Ext_ relative to αB_Pal_. Extrapolated to the context of oligomers of full-length protein therefore, the concurrent binding of two C-termini would precipitate the dissociation of a monomer, in a manner consistent with our previous NMR measurements [31,35,36].

### (c) A mass spectrometry assay to determine the thermodynamic and kinetic consequences of point-mutation

**Figure 5.**
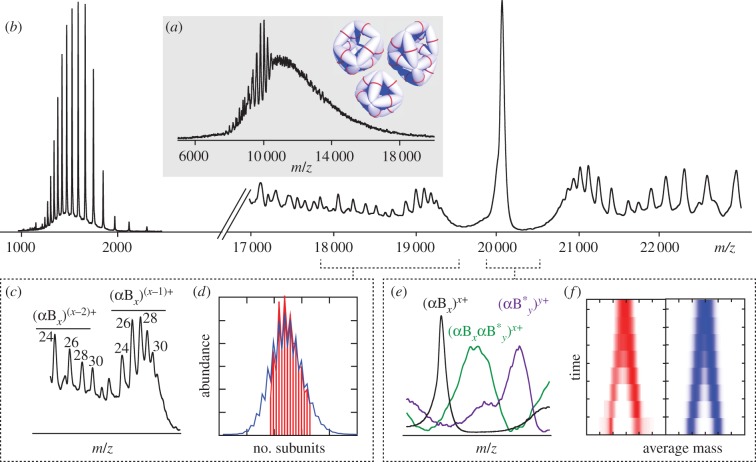
(*a*) Nanoelectrospray mass spectrum of the E156A/R157A double-mutant of full-length αB-crystallin, αB_ER_, under instrument conditions where non-covalent interactions are preserved. A broad range of unresolved signal is observed, indicative of the polydisperse ensemble of oligomers populated at equilibrium. (*b*) An equivalent spectrum obtained under activating instrument conditions, in which all the ions are subjected to energetic collisions with argon atoms. The peaks observed at high *m*/*z* correspond to αB_ER_ oligomers from which monomers have been dissociated, and are sufficiently resolved to allow the identification of the individual ‘stripped’ oligomers. (*c*) Expansion of the region 18 000–19 500 *m*/*z* allows the assignment of the different charge states to the stoichiometries, where *x* is the number of subunits in the oligomer. The value for *x* is indicated above each peak corresponding to even stoichiometries, with the lower abundance peaks stemming from oligomers with an odd number of subunits unlabelled. (*d*) From these data, the relative intensities of each stoichiometry can be extracted (red bars), and the best-fitting distribution according to a simple oligomerization model obtained (blue line) [65]. This allows us to determine the quantities *ΔG*_e_ and *ΔG*_d_, the inter- and intra-dimer free energies, respectively. (*e*) The peak at ≈20 100 *m*/*z* (black, (αB_*x*_)) corresponds to all αB_ER_ oligomers carrying the equivalent number of charges as subunits, and therefore is representative of the entire polydisperse ensemble. The peak for an isotopically labelled ^13^C equivalent (purple, (αB*_y_ )) is observed at higher *m*/*z*. Incubation of these two proteins results in the gradual disappearance of the homo-oligomers and the concomitant formation of hetero-oligomers (green, (αB_*x*_)(αB*_y_ )). (*e*) By monitoring the time-course of this subunit exchange reaction, the quaternary dynamics can be quantified. A ‘top-down’ view of the time-course (red, left), which shows the homo-oligomers coalescing into a distribution of hetero-oligomers, can be compared to simulated time-courses to obtain the best fit (blue, right), and thereby the rate constants *k*_e_^−^ and *k*_e+d_^−^ [65]. (Online version in colour.)

To further analyse the interactions between the C-terminus and the α-crystallin domain in the context of the oligomer, we performed an alanine scan, generating a series of point mutants of full-length αB-crystallin that collectively act to encompass the entire palindromic region of sequence ^156^ERTIPITRE^164^ (figure 1), and examined them by means of MS. Our method has been detailed previously [65], and is described briefly below. Using appropriate ion generation and transmission conditions [71], the intact αB-crystallin oligomers are directly measured in the mass spectrometer. The resultant mass spectra feature a broad region of signal arising from a large number of stoichiometries with many overlapping charge states [19], that is essentially uninterpretable (figure 5*a*). To overcome this, we use a collisional activation strategy in which the oligomer ions are heated by successive collisions with a target gas until they dissociate [72]. The general mechanism of gas-phase dissociation dictates that oligomers dissociate into highly charged monomers (figure 5*b*, 1000–2000 *m*/*z*) and complementary ‘stripped oligomers’ (figure 5*b*, 16 000–24 000 *m*/*z*) [66]. From these data, we can assign the different peaks, extract their intensities, and thereby determine the oligomeric distribution of the αB-crystallin ensemble (figure 5*c*). This approach is cross-validated by the faithful back-calculation of the original mass spectrum [33,55]. Further verification comes from noting that, while of much higher resolution, this distribution matches that measured by using solution phase methods extremely well [48].

Although αB-crystallin populates many stoichiometries, their distribution can be well described using a relatively simple oligomerization model [65]. This model invokes only two interactions between individual αB-crystallin monomers, corresponding to intra-dimer (dimer, d) and inter-dimer (edge, e) interfaces (see schematic in the electronic supplementary material, table S1). This two-parameter model assumes individual oligomers are in dynamic equilibrium with their corresponding monomers, an assumption justified by the appearance of oligomers comprising an odd number of subunits in experimental data and in facile subunit exchange [65]. The model predicts that the basic monomeric structure is identical in all oligomers, which is confirmed by NMR experiments [25,31], and that the dimer interface is labile, as suggested by our results on the core domain (figure 2) and previous measurements [23,68,73]. Through fitting this model to the MS data, the oligomeric distributions can be reproduced accurately, and the association free energies of the edge and dimer interfaces (*ΔG*_d_, and *ΔG*_e_, respectively) quantified (figure 2*d*) [65].

To complement these thermodynamic parameters, the rate constants that govern the inter-conversion of the αB-crystallin oligomers can be determined using subunit exchange reactions in which the protein is incubated with a labelled counterpart [74]. This is illustrated schematically for the reaction between αB-crystallin and its ^13^C equivalent (figure 5*e*). The peak at ≈20 100 *m*/*z* (figure 5*e*, black) comprises αB-crystallin-stripped oligomers, each carrying as many charges as subunits, and is representative of all oligomers that contribute to the ensemble [19]. Mass spectra of the ^13^C protein reveal the same oligomeric distribution as the ^12^C counterparts, but, due to the additional mass, the equivalent peak appears at ≈21 000 *m*/*z* (figure 5*e*, purple). Upon incubation, the two peaks corresponding to homo-oligomers coalescence into one broad peak centred on their midpoint (figure 5*e*, green), indicating the formation of hetero-oligomers [38]. This subunit exchange can be monitored by collecting data at different time points (figure 5*f*, red), and fitting these data to simulated time courses generated using our oligomerization model (figure 5*f*, blue) allows the extraction of the corresponding rate constants [65].

### (d) Destabilization of the C-terminal interaction stabilizes the intra-dimer interfaces

**Figure 6.**
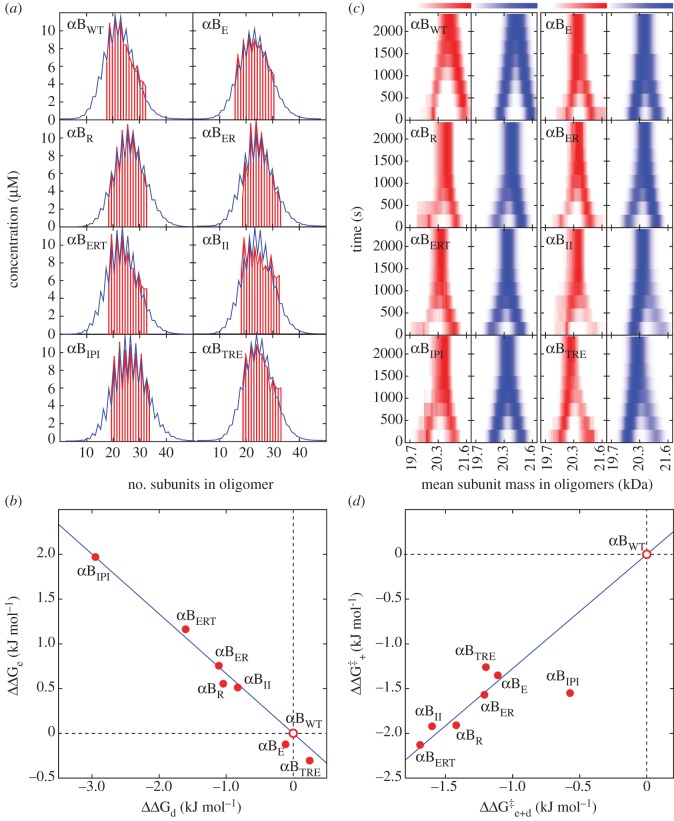
(*a*) Oligomeric distributions of the different αB-crystallin mutants, determined as described in figure 5*a–d*. Distributions were obtained for all proteins at 37°C, pH 6.9, in 200 mM ammonium acetate, and were normalized to the total protein concentration. All the proteins are polydisperse, centred on 24–28 subunits. Notably, the proportion of even and odd stoichiometries varies relative to αB_WT_, resulting in either increased ‘spikiness’ or smoothness of the distribution. For example, αB_IPI_ is considerably spikier than αB_WT_, indicative of a stronger intra-dimer interface, i.e. a more negative *ΔG*_d_. (*b*) Plotting the change in association free energy upon mutation (*ΔΔG* = *ΔG*_mutant_ − *ΔG*_WT_; positive value corresponds to destabilizing mutation) of the inter-dimer (*x*-axis, *ΔΔG*_e_) and intra-dimer (*y*-axis, *ΔΔG*_d_) interface for our alanine mutations shows a clear negative correlation. This reveals that as one interface is strengthened the other is weakened. The sum of the two quantities *ΔΔG*_e+d_ is found to be almost zero in all cases (see the electronic supplementary material, table S1). (*c*) The subunit exchange data for the same proteins, obtained at 37°C, pH 6.9, in 200 mM ammonium acetate. While the reaction takes approx 2500 s to complete for αB_WT_, all mutants exchange considerably faster. By comparing the determined rate constants we can extract the change in free energy of activation upon mutation (*ΔΔG*^‡^ = *ΔG*^‡^_mutant_ − *ΔG*^‡^_WT_; negative value corresponds to rate-enhancing mutation), for both association (*y*-axis, *ΔΔG*_+_^‡^) and dissociation of the combined intra- and inter-dimer interfaces (*x*-axis, *ΔΔG*_e+d_^‡^). As with the thermodynamic quantities shown in (*b*), the changes in activation free energy of the forward and backward rates are tightly correlated. (Online version in colour.)

We obtained the oligomer distributions for the wild-type protein and the seven different mutants (nomenclature given in figure 1) at pH 7 and 37 °C. In all cases, the proteins assemble into polydisperse ensembles centred around a 24–28 mer (figure 6*a*), consistent with previous measurements for αB_WT_ and phosphorylated versions [65,75]. In addition, all of the distributions were found to have a ‘saw-toothed’ character, with oligomers comprising an even number of subunits more abundant than those composed of an odd number. Significantly, the extent of this disparity varies according to the location of the mutation: for example, the αB_IPI_ mutant is ‘spikier’ than αB_WT._ In the context of the model, increased disparity is diagnostic of a strengthening of the dimer interface, and vice versa.

In order to quantify this effect, we determined *ΔG*_e_ and *ΔG*_d_ values for each of the proteins (see the electronic supplementary material, table S1). In order to readily compare the relative effects of the mutations, we calculated the corresponding *ΔΔG*s, where a positive value suggests that the mutant is less stable than the wild-type, and a negative value the converse (see the electronic supplementary material, table S1). A plot of *ΔΔG*_d_ versus *ΔΔG*_e_ reveals a clear negative correlation of these two quantities (figure 6*b*). The majority of the mutations cause a weakening of the edge interface, of up to 2 kJ mol^−1^. Remarkably, we observe a concomitant and compensatory strengthening of the dimer interfaces. This demonstrates a clear allosteric coupling between the two interfaces [76]. Tighter binding of the C-terminal tail to the β4+8 groove weakens the interaction across the intra-dimer interface formed by the β6–7 strands, and vice versa. This finding is consistent with our titration data on αB_Dom_, where we show that binding the second petide to the dimer destabilizes the dimer interface. Furthermore, our results reveal that residues upstream of I161 have significant effects on the distribution, whereas those downstream of I161 (as exemplified by αB_TRE_) have negligible consequences on the distribution.

Structures of isolated αB-crystallin dimers have been determined in which the C-terminus is bound into the β4–8 groove. It is possible to estimate the difference in free energy upon point mutation *in silico*, allowing us to obtain theoretical *ΔΔG*_e_ values to compare with our data (see the electronic supplementary material, table S1). While there is some qualitative correspondence in that most of the mutations are predicted to destabilize the interaction both *in silico* and *in vitro*, quantitatively there are dramatic differences in the magnitude of the values and discrepancies as to which residues have the greatest contribution. Most notably, the *in silico* calculations on the bound state predict that the mutations to the IXI region should cause a destabilization of almost 40 kJ mol^−1^, an effect approximately 30-fold greater than that measured experimentally. This apparent conflict might however be rationalized by the IXI populating a bound state to the order of only a few per cent, consistent with solution-state NMR experiments [31,38].

Furthermore, the calculations predict the αB_TRE_ mutation to cause a significant destabilization of the interaction. By contrast, we see no change in the free energy, within the limits of experimental error, for this triple-mutant. Taken together, we can conclude that the free energies predicted for the bound state of the C-terminal tail are very far removed from that measured in solution. By contrast, the calculations are broadly consistent with our, and others' [69], titration experiments on αB_Dom_ that reveal *K*_D_s in the 70–300 μM range; and our NMR measurements that show that in the oligomers the tail is largely present in a disordered, and unbound conformation [31,38].

### (e) C-terminal mutations cause an increase in both dissociation and association rates

In order to complement our measurement of the thermodynamic consequences of the mutations, subunit exchange experiments were performed to obtain the monomer association and dissociation rates. Incubations between unlabelled and ^13^C protein were monitored as described above, and the time taken for the respective homo-oligomers to equilibrate into a distribution of hetero-oligomers was determined. For αB_WT_, this process was complete after approximately 40 min (figure 6*c*). By contrast, all the mutants exchanged significantly faster, with the fastest, αB_ERT_, reaching equilibrium within 20 min (figure 6*c*). This demonstrates that mutations in the C-terminal region affect not only the strength of the quaternary interfaces within αB-crystallin, but also their associated dynamics.

The difference in subunit exchange rates was quantified by extracting the dissociation rate constants *k*^−^_e_ and *k*^−^_e + d_ and pseudo-first-order association rate *k*^+^[*α*B_1_], where [*α*B_1_] is the concentration of free monomer in solution (see the electronic supplementary material, table S1). In the case of the mutations studied here, all display both increased association and dissociation rates. To enable comparison between the mutant and the wild-type, we calculated *ΔΔG*^‡^ values (change in the free energy of activation) in each case. In all cases, the *ΔΔG*^‡^s are significantly negative and, similar to the finding with the thermodynamic data (figure 6*b*), *ΔΔG*_+_^‡^ (association) and *ΔΔG*_e+d_^‡^ are also strongly correlated (figure 6*d*). In other words, all mutations that increase the rate of association increase the rate of dissociation by approximately the same amount. Similar to the result obtained for the thermodynamic parameters, mutations upstream of I161 were found to have the most significant effects.

## 4. Discussion

We have presented a detailed investigation on the influence of the palindromic C-terminal residues on the thermodynamics and kinetics of αB-crystallin oligomers. We have addressed the problem using two orthogonal approaches. First, we performed titration experiments between peptides mimicking the C-terminal region of αB-crystallin and αB_Dom_, determined their binding affinities, and noted effects on the intra-dimer interface induced by binding. Second, we mutated residues in the C-terminal tail and observed the thermodynamic and kinetic consequences on the oligomers. Both approaches give results that are internally consistent and enable a deeper understanding of the molecular interactions that dictate the properties of αB-crystallin oligomers.

### (a) Mass spectrometry for studying the thermodynamics and kinetics of protein interfaces

We employed an IM–MS strategy to analyse titrations of αB_Dom_ and C-terminal mimicking peptides to robustly quantify the different molecular species that coexist in solution. This allowed us to extract the *K*_D_s of peptide binding and, rather than providing an ensemble average over the stoichiometries present at equilibrium, do so for both the monomeric and dimeric forms of αB_Dom_. While quantitatively consistent with ensemble measurements from NMR [69], interestingly we observe that two sequential peptide binding events to (αB_Dom_)_2_ are not equivalent. While binding of the first peptide is itself weak, it is not only significantly more difficult to bind the second but there is also a concomitant destabilization of the dimer interface. This effect is particularly pronounced in the case of αB_Ext_, which is longer than αB_Pal_, and has a significant number of positively charged residues at the C-terminus. Both steric and coulombic interactions are therefore likely to play a role in decreasing the binding affinity of the second peptide. As such, while one might intuitively expect the high local concentration of C-terminal tail in the oligomer to effectively overcome its weak binding affinity [69], our results indicate that additional tail-binding events are less favourable and precipitate dissociation of the oligomer. This is supported by the observation that not only does truncation of the C-terminus reduce the rate of subunit exchange of the α-crystallins [38,39], but also that mutating charged residues affects their self-assembly [40]. Binding of the second peptide has an additional interesting effect in that the intra-dimer interface is destabilized. This remarkable finding is consistent with the model derived by solution-state NMR that suggests that subunit exchange is facilitated by the C-terminus binding the β4+8 groove [31,36].

To complement studies on the truncated αB-crystallin construct, we examined point mutants of the full-length protein. The effects of individual and multiple alanine mutations on both thermodynamic and kinetic properties of the oligomers were then quantified by means of MS. The advantages of MS for monitoring the quaternary consequences of such alanine-scanning rest in the high resolution of separation in both mass and time afforded by the approach [48]. As a result, in this study, we have successfully extracted the changes in rate constants and free energies using experiments performed on the minute time-scale.

### (b) The inter- and intra-dimer interfaces of αB-crystallin are allosterically coupled and energy-compensate to maintain oligomer size

Our study has revealed that while the average oligomer size was essentially unchanged by mutation, the individual interactions that define the assembly are significantly impacted. We find that destabilization of the edge interface caused by alanine mutation results in a stabilization of the intra-dimer interface. This finding shows that the effect of binding the C-terminus at a first site (in this case, the β4–β8 groove) influences the interactions at a second, distal site (the β6–7 dimer interface): the hallmark of allosteric communication within the protein [76]. This phenomenon was previously noted in experiments examining the influence of pH on the distribution of wild-type αB-crystallin [65]. Similarly, phosphorylation of the N-terminus, which weakens the dimer interface [75,77], likely by binding a cleft on the inside of the oligomer [22], results in a strengthening of the edge interaction [65]. This role of the β4–β8 groove and C-terminal tail in allosteric communication rationalizes their identification as important regulatory regions in the molecular chaperone function of αB-crystallin [78–81].

Taken together, while the inter- and intra-dimer free energies can vary substantially, the effect of this energy compensation is such that the quantity *Δ*G_e+d_ is kept approximately constant (figure 6*b*). This is the value that predominantly dictates the average oligomer size [65], revealing that αB-crystallin oligomers have the ability to radically alter their interface dynamics, as well as structure [23], yet retain an essentially constant gross oligomeric distribution. Functionally, this is likely to be an important property for maintaining eye-lens transparency at high protein concentration while avoiding crystallization [82]. In addition, the molecular chaperone activity of sHSPs may itself benefit from such polydispersity, through the provision of a diversity of binding surfaces for intercepting a wide range of destabilized target proteins [83]. With polydispersity potentially crucial to both these roles of αB-crystallin in the body, it is perhaps unsurprising that the vast number of mutations reported in the literature have had only a limited effect on the overall oligomerization of the protein, and the quest for a homogeneous quaternary structure remains unfulfilled [18].

### (c) The synergistic roles of the C-terminal tail, extension and palindrome

Our experiments have revealed the significant contributions made to the oligomeric dynamics and interface stabilities by residues in the C-terminal region of αB-crystallin. It is notable that none of the mutations resulted in complete disassembly of the oligomer, which, together with evidence that truncated constructs of the α-crystallins remain assembly competent [23], reveals that the C-terminus is not the sole provider of thermodynamic stability to the oligomer. Instead, its role seems to be more subtle, acting as a ‘gate-keeper’ for the quaternary dynamics of the protein [31].

We have demonstrated here that all of the mutations on the C-terminal tail have small but measurable effects (*ΔΔG* < 2 kJ mol^−1^) on the strength of the edge interface and the corresponding rates of association and dissociation. The effect of these changes is to modulate the proportion of time during which the C-terminal IXI remains bound to the oligomer. This provides a biophysical rationale as to why mutation or removal of C-terminal residues leads to aberrant function of αB-crystallin *in vitro* [32,40], in cells [41,42], and have been identified in dilated cardiomyopathy [44,45] and cataract [43,46]. Furthermore, a recent report on the monodisperse archaeal HSP14.0 apparently indicated a similarly transient C-terminal interaction [30], which raises the possibility that a regulatory role for this region of sequence may be widespread in the sHSP family.

**Figure 7.**
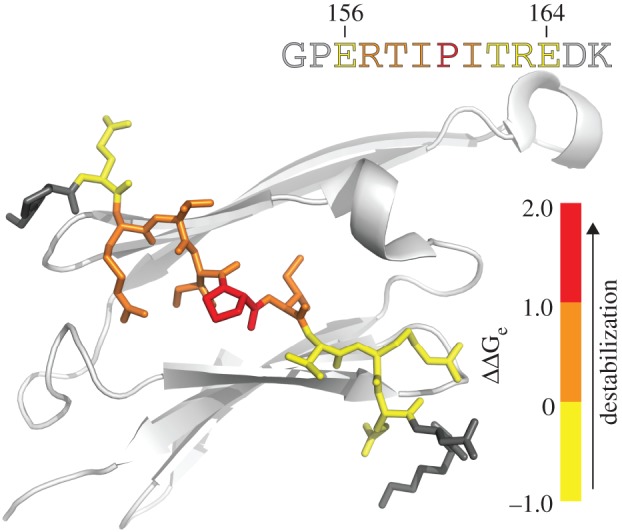
From the thermodynamic and kinetic data obtained from alanine mutations of αB-crystallin, we can obtain the relative contribution that each residue makes to the various *ΔΔG* values we obtain. The residue-specific *ΔΔG*_e_ values are shown here, projected onto the crystal structure of αB-crystallin (PDB: 3L1G). From these data, it is immediately apparent that mutations of residues R157 to I161 have significant effects, whereas mutations of E156 and T162–E164 much less so. Although shown here for *ΔΔG*_e_, the same trend is present in all of the thermodynamic and kinetic parameters considered (see the electronic supplementary material, table S1). The alanine mutations taken together allow identification of the individual contributions of interacting residues in αB-crystallin oligomers. (Online version in colour.)

While mutations upstream of I161 were found to significantly destabilize the inter-dimer interface, mutations downstream had no discernable effects on the distribution (figure 7). Truncated α-crystallin constructs have been crystallized with the C-terminus in two different orientations, facilitated by its palindromic nature [23,24]. Our data show that while mutation of the ERT before the IXI has significant effects on the distribution, mutation of the TRE following the IXI does not, suggesting that in solution, the oligomers probably have a significantly preferred orientation. This is in line with previous NMR results that have demonstrated the extension to tumble freely in solution [84], even in homogenates mimicking the crowded environment of the eye lens [85] or solid-state NMR preparations [36]. Furthermore, we have found that the *K*_D_ of the αB_Ext_ is significantly higher than that of αB_Pal_, demonstrating that the extension acts to facilitate the detachment of the tail. This acts to directly influence the rate of subunit exchange, demonstrating why mutant proteins with shorter extensions have reduced quaternary dynamics [38,39].

## 5. Conclusions

Our study demonstrates that MS can be used to provide a highly detailed view of the macroscopic quaternary structure and dynamics of αB-crystallin. Moreover, they strongly support a model we recently proposed to explain many thermodynamic and kinetic properties of the oligomers. We show in both truncated αB-crystallin constructs and full-length oligomers that there is allosteric communication between the β4–8 groove and the β6+7 dimer interface. When two C-terminal peptides bind, the dimer interface is significantly destabilized, thus facilitating monomer dissociation and subunit exchange, and potentially exposing target binding sites. Moreover, mutational studies reveal that when tail-to-groove interactions are destabilized there is a corresponding increase in strength in the dimer interface within the oligomers and an increase in the rates at which monomers associate and dissociate. These observations provide a rationale as to why variant proteins with mutations in this region are associated with multiple disease states. Furthermore, our MS approach allows the quantification of the quaternary dynamics and oligomerization of a polydisperse protein, which not only opens the door for assessing the consequences of disease-related point mutants but also screening for small molecules that may act to prevent or reverse this.
